# Modulation of Electrocortical Brain Activity by Attention in Individuals with and without Tinnitus

**DOI:** 10.1155/2014/127824

**Published:** 2014-06-12

**Authors:** Brandon T. Paul, Ian C. Bruce, Daniel J. Bosnyak, David C. Thompson, Larry E. Roberts

**Affiliations:** ^1^Department of Psychology, Neuroscience & Behaviour, McMaster University, 1280 Main Street West, Hamilton, ON, Canada L8S 4K1; ^2^Department of Electrical and Computer Engineering, McMaster University, 1280 Main Street West, Hamilton, ON, Canada L8S 4K1; ^3^McMaster Institute for Music and the Mind, McMaster University, 1280 Main Street West, Hamilton, ON, Canada L8S 4K1

## Abstract

Age and hearing-level matched tinnitus and control groups were presented with a 40 Hz AM sound using a carrier frequency of either 5 kHz (in the tinnitus frequency region of the tinnitus subjects) or 500 Hz (below this region). On attended blocks subjects pressed a button after each sound indicating whether a single 40 Hz AM pulse of variable increased amplitude (target, probability 0.67) had or had not occurred. On passive blocks subjects rested and ignored the sounds. The amplitude of the 40 Hz auditory steady-state response (ASSR) localizing to primary auditory cortex (A1) increased with attention in control groups probed at 500 Hz and 5 kHz and in the tinnitus group probed at 500 Hz, but not in the tinnitus group probed at 5 kHz (128 channel EEG). N1 amplitude (this response localizing to nonprimary cortex, A2) increased with attention at both sound frequencies in controls but at neither frequency in tinnitus. We suggest that tinnitus-related neural activity occurring in the 5 kHz but not the 500 Hz region of tonotopic A1 disrupted attentional modulation of the 5 kHz ASSR in tinnitus subjects, while tinnitus-related activity in A1 distributing nontonotopically in A2 impaired modulation of N1 at both sound frequencies.

## 1. Introduction

Forms of neural plasticity are expressed by many neurons in central auditory structures and are believed to sculpt the neural changes that underlie the development of tinnitus and hyperacusis associated with hearing loss [1, 2]. Examples of neural changes attributed to neural plasticity in animal models include upregulation of somatosensory inputs to principal neurons in the dorsal cochlear nucleus (DCN) following section of the cochlear nerve [3] and broadening of the temporal integration window of spike-timing dependent plasticity for neurons in the DCN [4] and auditory cortex [5] in animals exhibiting behavioral evidence of tinnitus. Neural changes taking place after deafferentation may in turn affect how neural activity is modified when auditory training is applied to individuals with tinnitus, as is done by sound therapies intended to treat this condition. Roberts et al. [6] trained individuals with tinnitus and age and hearing-level matched controls to detect an auditory target embedded in a 5 kHz 40 Hz amplitude modulated (AM) sound. The 5 kHz 40 Hz AM sound was in the tinnitus frequency region (TFR) of the tinnitus subjects and evoked the stimulus-driven 40 Hz auditory steady response (ASSR) known to localize to sources in primary auditory cortex (A1) [7–10]. In agreement with earlier results obtained from normal hearing subjects [11, 12], the phase of the ASSR phase (the time delay between the 40 Hz stimulus and response waveforms) decreased progressively over training sessions in the control group, but ASSR phase did not change in the tinnitus group. In contrast, the amplitude of the ASSR (which was known from earlier research to be resistant to change) did not increase with training in controls, but ASSR amplitude increased with training in the tinnitus group, as did online ratings of the loudness of their tinnitus percept. It was suggested that abnormal synchronous neural activity underlying the tinnitus percept may have obstructed changes in ASSR phase in the tinnitus group, whereas reduced inhibition in A1 associated with tinnitus may have permitted an expansion of the cortical representation for 5 kHz that was prevented by competitive interactions within the tonotopic map of control subjects without tinnitus [6].

These results suggest that the effects of plasticity are modified in tinnitus sufferers by tinnitus-related neural activity occurring in auditory pathways. Further findings of Roberts et al. [6] suggested that effects of attention on neural responses are also modified in the tinnitus brain. In hearing-intact animals, neural plasticity is modulated by subcortical cholinergic and other neuromodulatory systems that receive top-down input from prefrontal cortex and project widely to the neocortex where they perform an attention-like function, making neurons more sensitive to their afferent inputs [13–17]. These mechanisms may account for the observation in normal hearing humans that auditory tasks that require top-down attention increase not only the amplitude of the ASSR localizing to A1, but also the amplitude of the N1 transient response whose cortical sources localize to secondary auditory cortex (A2) in the region of the planum temporale [7, 18]. In agreement with results obtained in normal hearing subjects, control subjects in the study of Roberts et al. [6] showed increased ASSR and N1 amplitude on active trials where detection of targets was required, compared to a passive condition where subjects were told to ignore the sounds and rest until the next active block was presented. However, modulation of ASSR and N1 amplitude by attention was abolished in the tinnitus group for N1 in all sessions of training and for ASSR amplitude on the first session with a weak modulation appearing subsequently as ASSR amplitude increased over trials. The results suggested that, although the top-down auditory attention system may work normally in tinnitus, its expression was obstructed by tinnitus-related neural activity occurring in the TFR of the tinnitus group where the sound to be detected (a 40 Hz AM 5 kHz carrier frequency) was located.

The present experiment evaluated this hypothesis by determining whether deficient modulation of ASSR and N1 amplitude by attention is observed when subjects with tinnitus are required to detect auditory targets embedded in a 40 Hz AM carrier of 500 Hz, which is well below the region where tinnitus-related neural activity is expected to occur. The results were compared in a unified analysis to the 5 kHz groups reported by Roberts et al. [6] which performed the same auditory detection task except for the carrier frequency chosen. In addition, two additional long-latency responses, namely, the N2 transient response (latency ~325 ms) and the auditory sustained response (SR, commencing after N2 and persisting to the end of stimulation), were studied in both groups, to determine whether modulation of these late responses was similarly affected by tinnitus.

## 2. Methods

### 2.1. Participants and Design

60 subjects (30 tinnitus and 30 controls) were recruited via McMaster University faculty and staff by email list servers and from our laboratory archive. One control subject was excluded from analysis due to noise in the electroencephalogram (EEG) that could not meet the requirement for artifact rejection in offline processing. Two further controls withdrew for unrelated medical reasons. Two tinnitus participants withdrew after expressing concern that the procedures might worsen their tinnitus. Of the remaining 55 subjects, 22 completed the 5 kHz study of Roberts et al. [6] and 33 subjects were new recruits assigned to 500 Hz and tested here. No subjects in the total sample reported use of medication during the time of the study; controls reported no history of tinnitus or ear diseases. Participants received an honorarium of $10 CAD per hour as well as reimbursement for parking fees. Subjects provided informed consent using procedures approved by the Research Ethics Board of McMaster University and consistent with the Declaration of Helsinki.

Tinnitus subjects completed a preliminary interview (intake session, about 90 minutes) that collected detailed information on personal history of their tinnitus. The Tinnitus Handicap Questionnaire (THQ) was administered to assess tinnitus attributes and impact on quality of life [19]. Pure-tone audiometric thresholds were measured using a GSI 61 audiometer with Telephonics 296 D200 (0.125–8.0 kHz) and Sennheiser HDA 200 (8.0–16 kHz) headphones using the pulsed-tone method. Properties of tinnitus were measured by computerized tools described by Roberts et al. [12]. Using the tools, subjects first identified the ear of tinnitus (left, right, or both) and tinnitus bandwidth (tonal, ringing, or hissing) following which they rated tinnitus loudness on a Borg CR100 visual analog scale. Next, subjects adjusted the loudness of each of 11 pure tones between 0.5 and 12.0 kHz to equal that of their tinnitus. The tinnitus frequency spectrum (likeness rating) was then taken for the same pure tones at the determined loudness level, followed by a brief test for residual inhibition. Control subjects completed the same intake procedure as tinnitus subjects except for procedures pertaining to tinnitus.

**Figure 1:**
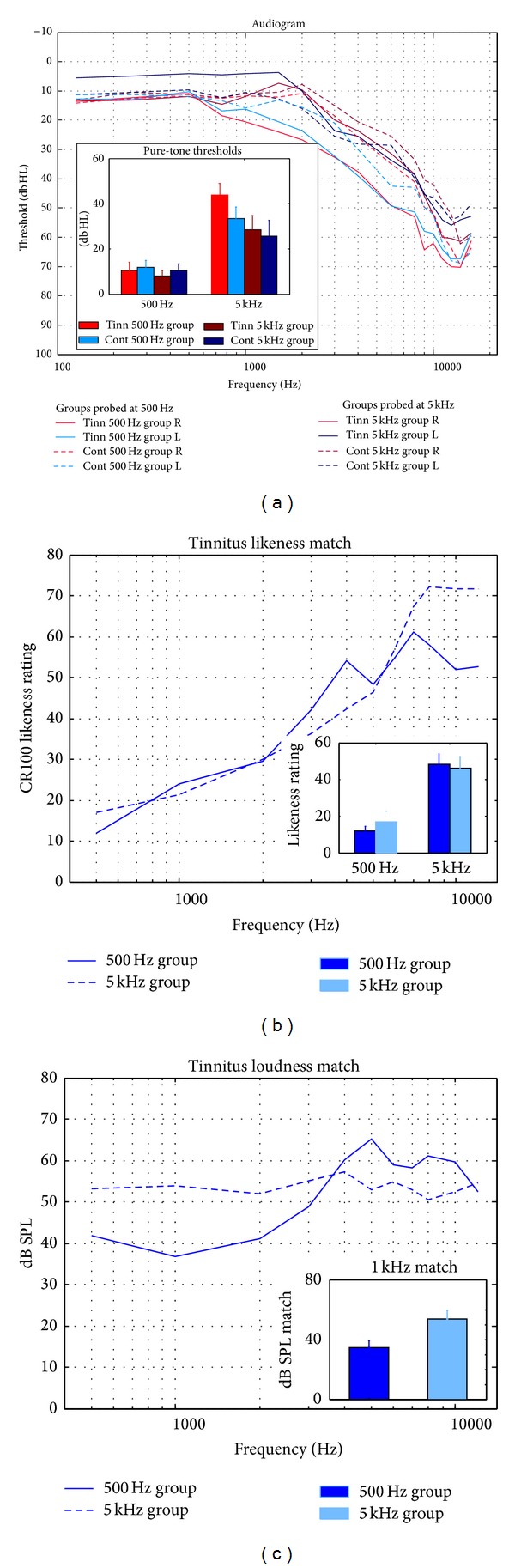
Audiogram, tinnitus spectrum, and tinnitus loudness matches. (a) Pure-tone audiograms (pulsed-tone method) from 0.125 to 16 kHz showing each ear and group separately. Comparisons of thresholds averaged across ears at 500 Hz and 5 kHz are shown in the inset bar graph (5 kHz interpolated between 4 kHz and 6 kHz) separately for groups probed with 500 Hz and 5 kHz sounds. (b) Tinnitus likeness ratings from 0.5 to 12 kHz for both tinnitus groups and an inset bar graph comparing 500 Hz ratings to 5 kHz ratings in each group. 500 Hz ratings are below the tinnitus spectrum which commences above a likeness rating of 40 (a sound beginning to resemble tinnitus; Roberts et al. 2008). (c) Tinnitus loudness matches from 0.5 to 12 kHz for both tinnitus groups. Inset bar graphs compare loudness matches at a common 1 kHz frequency.

Four groups of subjects were studied: controls tested at 500 Hz (Cont500 Hz), tinnitus subjects at 500 Hz (Tinn500 Hz), control subjects at 5 kHz (Cont5 kHz), and tinnitus subjects at 5 kHz (Tinn5 kHz). The tinnitus and control groups were matched for age within the two stimulus frequencies and as much as possible between the two frequencies. The number, age, and gender of subjects in each group and the sound levels experienced by the subjects are given in Table 1 where properties of tinnitus are also reported for the two tinnitus groups.  Figure 1 shows audiometric thresholds for each group and, for tinnitus subjects only, the tinnitus spectrum and loudness matches for sound frequencies between 500 Hz and 12 kHz. Approximately one week lapsed between the intake session and the experimental session described next.

### 2.2. Stimuli

The stimuli were 500 Hz and 5 kHz pure tones AM by a 40.96 Hz sinusoid (called 40 Hz, 100% modulation depth following the modulation wave). Tone duration was 975.56 ms, such that each stimulus contained 40 AM pulses. Stimuli were generated by a digital signal processor (Tucker-Davis RP.2) and presented binaurally through ear inserts (Etymotic ER2). Sound levels were determined by a loudness matching paradigm in which subjects in the 500 Hz groups matched the loudness of the stimulus to a reference pure tone of 1 kHz presented at 65 dB SL and subjects in the 5 kHz groups to a reference tone of 2 kHz presented at 65 dB SPL. These matching procedures aligned the groups with those of earlier research [2, 6] and equated subjective stimulus loudness between the tinnitus and control groups at each probe frequency. However, it was inevitable that probe intensity measured in SPL would vary between the 500 Hz and 5 kHz groups as a consequence of threshold shifts at 5 kHz and hyperacusis in the tinnitus groups. Possible effects of probe intensity were evaluated by regressing effects of attention expressed in each brain response on probe intensity in SPL, which was known for each subject.

### 2.3. Auditory Task

**Figure 2:**
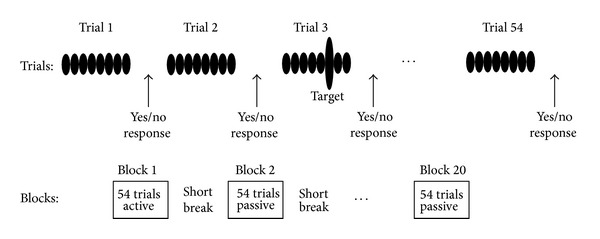
Auditory task. Upper panel: three standard 40 Hz AM stimuli and one target stimulus containing a single amplitude-enhanced AM pulse (target) are illustrated by cartoons containing 8 AM pulses (40 pulses were delivered on each trial). Approximately 2/3rd of the stimuli contained a target of variable enhanced amplitude such that not all targets were detectable. Lower panel: on active blocks participants identified whether a target was present or not; on passive blocks participants ignored the sounds and waited for the next active block. Blocks contained 54 trials and alternated between active and passive blocks for a total of 20 blocks per session.

The auditory task is described in Figure 2. Subjects sat in a sound-attenuated (ambient noise level 16 dBA SPL) and electrically shielded booth, comfortably in a chair distanced 1.4 m from a computer monitor. There were two types of stimuli: standard stimuli and stimuli containing a target. The two stimuli were identical except that target stimuli contained a single 40 Hz pulse of variable increased amplitude (target) that occurred randomly at 415 ms, 610 ms, or 805 ms after stimulus onset. Approximately 2/3rd of the stimuli contained a target; however, because approximately 1/3rd of the targets were expected to be below or close to the threshold of detection, target stimuli likely were heard on about 50% of trials. Trials of both types (standard and target) unfolded in either active blocks or passive blocks with each block containing 54 stimuli and lasting roughly 2.5 minutes. On active blocks, the word “Listen” appeared in a text box on the computer screen, instructing participants to attend to the trial for a target event. After stimulus completion text on the screen prompted, “Did you hear a target?” As per instructions on the screen, participants pressed the left mouse button “yes” if they had detected a target and a right mouse button “no” if they had not. Correct responses (hits and correct rejections) generated a green text box for 400 ms providing appropriate feedback. Incorrect responses (misses and false alarms) produced a red text box for the same duration. An intertrial interval (ITI) varying between 1400 and 1600 ms commenced with each behavioral response, giving a variable interval of about 1900 ms including the feedback cue and depending on behavioral response latency. During passive blocks, the text “Stop responding and ignore stimulus” appeared continuously on the computer screen, indicating that participants should ignore the sounds and wait until the next active block. The ITI was randomly varied between 1600 and 1900 ms (stimulus offset to onset) for passive blocks, to be comparable to active blocks. Each session began with an active block and alternated with passive blocks for a total of 20 blocks (10 active and 10 passive) with 54 trials in each (Figure 2).

It should be noted that active trials on this task not only required attention to the stimuli but also involved other cognitive functions such as processing of target events, behavioral response selection, and perhaps also anticipation of correctness feedback. Short latency responses such as the ASSR and N1 are likely to be dominated by attention since this process was necessarily deployed commencing at trial onset with other functions following after target detection. Consistent with this expectation, Gander et al. [7] found that attention modulated ASSR amplitude in a dual auditory-visual task when all other task requirements (processing of feedback events, response selection, and correctness feedback) were held constant. We refer herein to the active/passive manipulation as one affecting attention but acknowledge that long-latency brain responses in particular may reflect overlapping cognitive functions.

Immediately prior to the session, each subject completed a staircase procedure in order to determine a set of target amplitudes suitable for the detection task. 80 stimuli were presented each containing a target, commencing with a 200% amplitude increase known to be detectable by inexperienced subjects. Target amplitude decreased after each “yes” response and increased after each “no”; target amplitude at the end of 80 trials was taken as the amplitude corresponding to the subject's threshold of detection (TH). A set of six target stimuli was then generated for each subject consisting of TH, TH ± 5%, TH ± 10%, and TH + 20% for use on the detection task. TH varied between subjects and averaged 47% over all subjects.

### 2.4. Electrophysiological Recording

The EEG was recorded from a 128-channel Biosemi ActiveTwo amplifier (Cortech Solutions, Wilmington, NC) and sampled at 2048 Hz. Before recording, the electrode array positions were digitized for each participant (Polhemus Fastrak). EEG data were stored as continuous data files referenced to the vertex electrode.

### 2.5. Signal Processing

Eyeblink and other movement artifacts were removed from raw continuous data by the spatial filtering option of BESA (version 5.1.8; MEGIS Software GmbH, Grafelfing, Germany). Responses were epoched around 100 ms pre- and poststimulus baselines.

**Figure 3:**
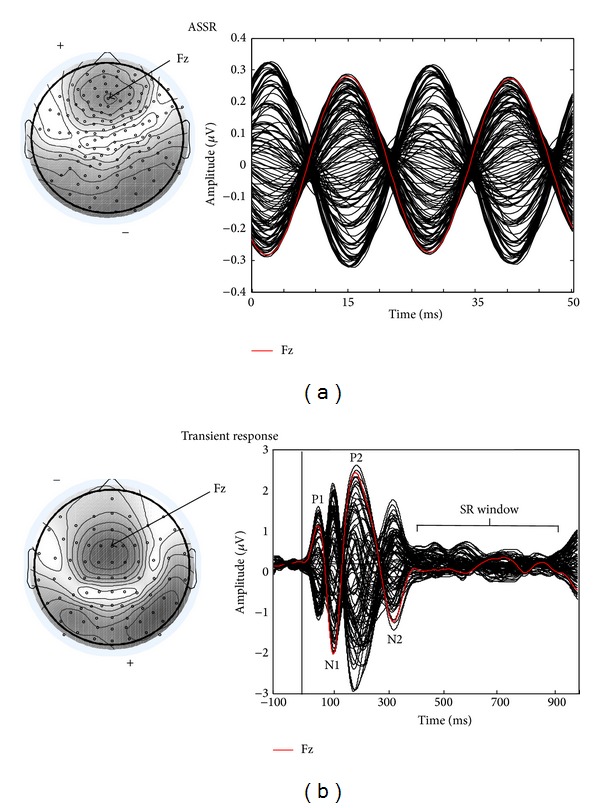
Representative topographies and time domain waveforms for the 40 Hz auditory steady-state response (ASSR) and N1 response derived from the grand average of active trials from control subjects probed with a 500 Hz stimulus. (a) shows the ASSR during the interval 244–952 ms poststimulus, collapsed down to two 40 Hz AM cycles. An alternating dipolar waveform is observed (one for each AM cycle). ASSR amplitude was calculated as the total field power of all electrodes in the two-cycle AM waveform. (b) A dipolar N1 is seen peaking at 100 ms poststimulus. N1 amplitude was calculated as total field power at the peak of the dipolar waveform. The transient responses P1, P2, and N2 and the time range for the auditory sustained response (SR) are also labeled in the waveform. For the purpose of visualization, the trace in the right panel is high pass filtered at 2 Hz to distinguish N2 from the SR which is attenuated as shown here. In each panel the Fz electrode is shown in red.

*40 Hz Steady-State Response. *EEG responses for ~85% of trials (rejecting trials with artifacts exceeding 100 *μ*V between 30 and 50 Hz) were used for analysis of the ASSR. Data were converted to the average reference and filtered 40 to 42 Hz (zero phase). For each of the 128 channels, data between 244 and 952 ms poststimulus were collapsed to a two-AM cycle average waveform for each subject (see Figure 3). Because the ASSR is reflected in most electrodes, ASSR amplitude was calculated as the total field power (TFP) determined by Fourier transform summed over 128 electrodes, following Gander et al. [20] and Roberts et al. [6].

*Transient Responses. *EEG responses for ~80% of trials (rejecting trials with artifacts exceeding 150 *μ*V between 1 and 20 Hz) were used for analysis of transient responses. Epoched data were averaged and interpolated to the 81-channel “reference free” average reference montage of BESA using each participant's digitized electrode array positions, which reduced individual differences in electrode cap placement between subjects. Data were then filtered from 0.2 to 20 Hz (zero phase). The latencies of P1 (from time window 30–85 ms), N1 (85–140 ms), P2 (140–230 ms), and N2 (250–350 ms) transient responses were identified from electrode Fz where the responses typically reached their amplitude maximum [7]. TFP for each response was calculated as the sum of each channel's squared voltage at the peak latency of electrode Fz (Figure 3). The auditory sustained response (SR) was calculated as the TFP over the time interval 400–900 ms (Figure 3). Two subjects (both in the 500 Hz tinnitus group) were omitted from the analysis of the SR because of the electrode drift exceeding −50 *μ*V past 400 ms.

### 2.6. Statistical Evaluation

Repeated measures ANOVAs were performed using the General Linear Model of Statistica (version 6.0). Least significant difference (LSD) tests were used to describe significant main effects and interactions. Group comparisons not addressed by ANOVA were evaluated by *t*-tests. Significance level was set at *α* = 0.05. Further details regarding statistical approach are reported where appropriate in Section 3.

## 3. Results

### 3.1. Behavioral Responses

**Figure 4:**
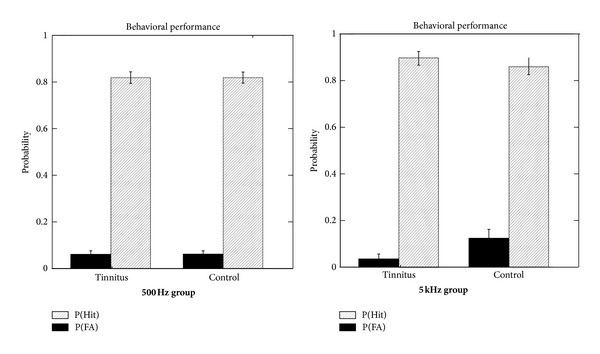
Performance on the behavioral task for both tinnitus and control groups probed at 500 and 5 kHz. The probability of a hit (*P*(Hit)) is averaged across the six target amplitude enhancements. The probability of a false alarm (*P*(FA)) was determined from trials with no target.

Performance on the behavioral task is presented in Figure 4. The probability of a hit (*P*(H)) exceeded the probability of a false alarm (*P*(FA)) for all subjects with no differences between the tinnitus and control groups or the two carrier frequencies on this measure. *P*(H) averaged 0.85 overall indicating that for most subjects at least one of the six target stimuli was not detectable.

### 3.2. Electrophysiological Responses

#### 3.2.1. Effects of Carrier Frequency and Group on Passive Blocks

In the first analysis, ANOVAs including the variables group (tinnitus/control) and frequency (500 Hz and 5 kHz) were applied to passive blocks for each brain response to identify effects of these variables on brain activity in the absence of attended performance. ANOVA returned main effects for carrier frequency (500 Hz versus 5 kHz groups) on these blocks for the ASSR (*F*(1,51) = 10.38, *P* = 0.002), P1 (*F*(1,51) = 11.87, *P* = 0.001), N1  (*F*(1,51) = 10.17, *P* = 0.002), P2 (*F*(1,51) = 12.93, *P* = 0.001), and the SR (*F*(1,49) = 5.31, *P* = 0.025), with similar results for N2 (*F*(1.51) = 2.40, *P* = 0.127). For each response TFP was larger in the 500 Hz groups than in the 5 kHz groups in accordance with the known dependence of the amplitude of the ASSR and transient responses on carrier frequency [21]. No main effects involving group reached significance for any response on passive blocks, although P2 tended to be larger in control subjects than in the tinnitus groups (*P* = 0.078) on these blocks. Interactions between carrier frequency and group did not reach significance for any response on passive blocks.

#### 3.2.2. Effects of Attention (Active versus Passive Blocks)

Effects of attention were evaluated first by comparing response TFP on active blocks where attention to the probe stimuli was required with that on passive blocks where subjects were instructed to ignore the stimuli and rest. No main effects or interactions involving active/passive were found for P1 and P2 responses, and these responses are not discussed further. However, the main effects of attention were found for the ASSR (*F*(1,51) = 10.38, *P* = 0.002), N1 (*F*(1,51) = 7.51, *P* = 0.008), N2 (*F*(1,51) = 29.12, *P* < 0.001), and the SR (*F*(1,49) = 28.71, *P* < 0.001).

Effects of attention on these responses were examined in more detail, as follows. For each subject and response, the effect of attention was calculated (1) as the difference in TFP between active and passive blocks (passive subtracted from active) and by (2) representing the attention effect as TFP on active trials divided by TFP on passive trials (this ratio minus 1, to represent no effect of attention as zero). Distributions of these measures (*n* = 55 subjects) were then examined for kurtosis, which can be pronounced for the ASSR where large but repeatable individual differences are known to occur (test-retest reliability *r* > 0.90, [2]), likely reflecting summation of ASSR fields across two tonotopic maps sharing a common low frequency border in Heschl's gyrus. For ASSR amplitude kurtosis was lower for the ratio measure (2.94) than for the difference measure (19.4), whereas the reverse was true for N1 (5.95/2.37), N2 (13.4/10.37), and the SR (9.50/2.64). Thus for the additional analyses reported below, effects of attention were analyzed as the ratio measure for the ASSR and as the difference in TFP between active and passive blocks for N1, N2, and the SR. Effects were evaluated statistically by *t*-tests and by ANOVA applied to these measures. In addition, the topography of TFP on active and passive blocks and the difference in TFP (active-minus passive) are shown for all responses.

**Figure 5:**
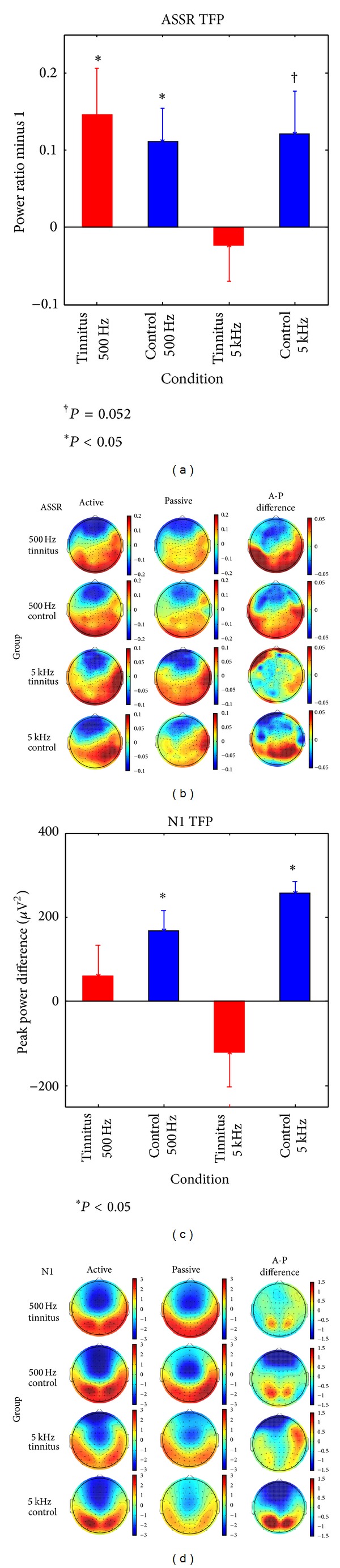
ASSR and N1 attention effects. (a) Effect of attention on ASSR TFP in each group (A/P TFP-1). (b) Voltage map of the ASSR taken at the time point of maximum total field power on active and passive blocks and the voltage difference map (active-passive blocks). (c) Effect of attention on N1 TFP in each group (active-passive blocks). (d) Active, passive, and difference voltage maps for N1 at the peak latency of electrode Fz. The error bars in (a) and (c) are one standard error (*P < 0.05; ^†^ *P* = 0.052).

*ASSR.* Effects of attention on the ASSR are shown in each group as TFP ratios in Figure 5(a) and as voltage difference maps in Figure 5(b). TFP ratios increased on active compared to passive blocks in the Cont500 Hz group (*t*(15) = 2.53, *P* = 0.023), Cont5 kHz group (*t*(10) = 2.199, *P* = 0.052), and in the Tinn500 Hz group (*t*(16) = 2.42, *P* = 0.028), but the TFP ratio did not increase on active blocks in the Tinn5k group (*t*(10) = −0.49, *P* = 0.628). This pattern can also be seen in the voltage difference maps presented for the four groups in Figure 5(b) (right column) where the voltage difference was minimal in the Tinn5 kHz condition. When the four groups were collapsed into one, the TFP ratio differed significantly from zero (*t*(54) = 3.54, *P* = 0.001) confirming the sensitivity of ASSR amplitude to attention. An ANOVA applied subsequently to TFP ratios with group and frequency as between-subjects variables found no significant effects, although the interaction of group and frequency approached significance (*F*(1,51) = 2.69, *P* = 0.106) reflecting the pattern seen in Figure 5(a). LSD tests within this interaction found the 5 kHz and 500 Hz tinnitus groups to be different from one another (*P* = 0.032) whereas contrasts of the Cont500 Hz group and the Cont5khz group to the Tinn5 kHz group reached *P* = 0.09 in each case. Effects of attention on ASSR amplitude were unrelated to ASSR amplitude on passive blocks when correlations were calculated between the two responses for the total sample (*r* = 0.09, *P* > 0.53) or for the tinnitus and control groups separately collapsing over probe frequency (*r*s = 0.24 and 0.09, resp., *P*s ≥ 0.21).

*N1*. Effects of attention on the N1 are shown for each group in Figure 5(c) (TFP difference between active and passive blocks) and in Figure 5(d) (voltage difference maps, right column). TFP increased on active compared to passive blocks in Cont500 Hz group (*t*(15) = 3.35, *P* = 0.043) and in the Cont5 kHz group (*t*(10) = 9.48, *P* < 0.001), but this difference did not reach significance in tinnitus groups probed at either frequency (*P*s > 0.17) notwithstanding a weak posterior modulation which can be seen in the voltage difference map for the Tinn500 Hz group. ANOVA applied to the difference in TFP between active and passive blocks returned the main effects of group (*F*(1,51) = 13.37, *P* = 0.001) and a significant interaction between group and frequency (*F*(1,51) = 4.12, *P* = 0.048). LSD tests within the interaction found that the N1 TFP difference was larger in the Cont5 kHz group than in either tinnitus condition (*P* < 0.04 or better) and also larger in the Cont500 Hz control group than in the Tinn5 kHz group (*P* < 0.004). Correlations between N1 TFP on passive blocks and the effect of attention on N1 TFP did not reach significance when the four groups were collapsed into a single sample (*r* = −0.23, *P* = 0.09) or when correlations were calculated for the tinnitus and control subjects separately collapsing over probe frequency (*r*s = −0.26 and −0.13, resp., *P*s ≥ 0.19).

**Figure 6:**
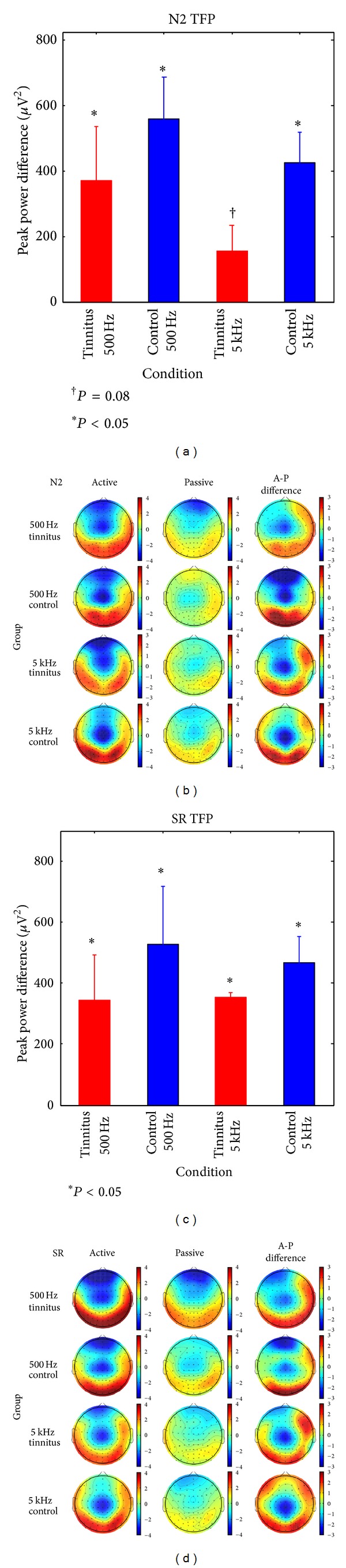
N2 and auditory SR scalp topography and attention effects. (a) Effect of attention on N2 TFP in each subject group (active-passive blocks). (b) Active, passive, and difference voltage maps for N2 at the peak latency of electrode Fz. (c) Effect of attention on SR TFP in each subject group (active-passive blocks). (d) Active, passive, and difference voltage maps for SR averaged from 400 to 900 ms. The error bars in (a) and (c) are one standard error (*P < 0.05; ^†^ *P* = 0.08).

*N2.* Effects of attention on N2 are shown for each group in Figure 6(a) (TFP difference measure) and as voltage difference maps in Figure 6(b) (right column). TFP increased on active compared to passive blocks in Cont500 Hz (*t*(15) = 4.42, *P* < 0.001), Cont5 kHz (*t*(10) = 4.47, *P* = 0.001), and Tinn500 Hz (*t*(16) = 2.21, *P* = 0.042) groups, while the difference in Tinn5 kHz approached significance (*t*(10) = 1.94, *P* = 0.081). Comparison of the groups by ANOVA found no significant main effects or interactions involving group or frequency, although the TFP difference between active and passive blocks tended to be larger in the control groups than in the tinnitus groups at both probe frequencies (main effect of group *P* = 0.105). The voltage maps of Figure 6(b) show further that N2 reached its maximum negativity at central electrodes, as did the TFP difference between active and passive blocks. This contrasts with the ASSR and N1 where amplitude maxima were focused frontocentrally on active trials (see Figures 5(b) and 5(d), resp.), particularly for the ASSR whose sources are localized tonotopically in the region of Heschl's gyrus.

*Sustained Response.* SR TFP increased on active compared to passive trials in all groups (Figure 6(c)). The results for each group were Tinn500 Hz (*t*(14) = 2.27, *P* = 0.039), Cont500 Hz (*t*(15) = 2.78, *P* = 0.0139), Tinn5 kHz (*t*(10) = 3.07, *P* = 0.012), and Cont5 kHz (*t*(10) = 5.46, *P* < 0.001). While active-passive differences in SR TFP tended to be larger in the control groups than in tinnitus, SR TFP differences for each group subjected to ANOVA revealed no main effects or interactions of group or frequency. On active blocks the SR showed a predominant negativity at central electrodes (Figure 6(d)) where the effect of attention was also predominantly expressed.

### 3.3. Demographics

The mean age of the subjects, their hearing thresholds at four sound frequencies, the intensity of the probe stimuli they received, and, where applicable, properties of their tinnitus are summarized for each group in Table 1. Correlations between several of these variables and (1) ASSR and N1 responses measured on passive blocks in the absence of attended performance and (2) effects of attention on ASSR and N1 TFP are reported in Table 2.

#### 3.3.1. Age

Subjects in the 500 Hz groups of Table 1 were on average 60.0 years old and those in the 5 kHz groups were 51.3 years old, a difference that was significant (*F*(1,51) = 8.33, *P* = 0.005). However, age range was similar among the four groups, and the tinnitus and control groups within each frequency were matched with no significant differences found in age between them. Age did not correlate significantly with ASSR and N1 responses measured on passive blocks or with effects of attention expressed in these responses when the tinnitus and control groups were collapsed at each frequency (Table 2).

#### 3.3.2. Hearing Thresholds

The audiograms for each group and ear measured to 16 kHz are reported in Figure 1(a). All groups exhibited thresholds exceeding 25 dB HL above 3 kHz while for the Tinn500 Hz group this criterion was met at 2 kHz. Threshold shifts were similar in both ears, with the only difference being thresholds about 7 dB greater in the right ear than in the left ear in the Tinn5 kHz group at the audiometric frequencies of 500 Hz and 1 kHz. To compare audiometric thresholds across all groups, 5 kHz thresholds were interpolated from 4 and 6 kHz thresholds, collapsed over left and right ears, and submitted to repeated-measures ANOVA with 500 Hz thresholds (see inset, Figure 1(a)). ANOVA returned the main effect of audiometric threshold frequency confirming higher thresholds at 5 kHz than 500 Hz in each subject group (*F*(1,51) = 66.23, *P* < 0.001). The main effect of group (tinnitus versus control) on 500 Hz and 5 kHz audiometric thresholds was not significant. Audiometric thresholds at 500 Hz and 5 kHz did not correlate with ASSR or N1 amplitude measured on passive blocks or with effects of attention in these responses when the tinnitus and control groups were collapsed at each frequency (Table 2).

#### 3.3.3. Probe Intensity

Probe intensity ranged from 47 to 93 dB SPL (*M* = 79.9) in the 500 Hz probe groups and from 40 to 74 dB SPL (*M* = 59.3) in the 5 kHz groups. Differences in probe SPL between the tinnitus and control groups tested at each carrier frequency averaged 2.5 dB or less (*P*s > 0.51), indicating that sound level matching between the groups was achieved within the 500 Hz and 5 kHz conditions. However, probe intensity collapsed over the tinnitus and control groups differed between the 500 Hz (80.0 dB SPL) and 5 kHz (59.2 dB SPL) conditions (*F*(1,51) = 73.05, *P* < 0.001). This difference was a function of several factors including a 15.7 dB HL threshold shift at 1 kHz in the 500 Hz groups (who would have experienced their 500 Hz probes at about 65 dB SL when matching to a 1 kHz 65 dB SL standard), a tendency for subjects to find 5 kHz 40 Hz AM sounds perceptually more salient than 500 Hz 40 Hz AM sounds, the presence of threshold shifts at 5 kHz in groups tested at this frequency, and some degree of unreported hyperacusis for a 5 kHz sound in the 5 kHz groups (which would have reduced probe SPL when matching a 65 dB SPL 2 kHz standard).

To assess whether probe intensity affected the brain responses, probe SPL was correlated with ASSR and N1 amplitude on passive blocks in the absence of attended performance and with effects of attention observed for these two responses. A correlation between probe level and ASSR amplitude was found on passive blocks in the 500 Hz group (*r*(31) = 0.55, *P* = 0.001; Table 2), indicating that louder 500 Hz probe stimuli evoked large ASSR responses in this group on passive trials. Probe intensity did not correlate significantly with ASSR responses evoked by 5 kHz probes or with N1 evoked by probes of either frequency on passive blocks. We also correlated probe intensity with effects of attention on ASSR and N1 amplitude collapsing the tinnitus and control groups within the 500 Hz and 5 kHz conditions. There was a weak tendency for stronger probe stimuli to be associated with larger effects of attention on ASSR amplitude in the 500 Hz groups (*r* = 0.32, *P* < 0.07), but no correlations between probe intensity and ASSR and N1 attention effects reached significance in the 500 Hz and 5 kHz conditions (see Table 2).

#### 3.3.4. Tinnitus Characteristics

The tinnitus likeness matches obtained in the Tinn500 Hz and Tinn5 kHz groups are shown in Figure 1(b) where a likeness rating of 40 indicates a sound that is beginning to resemble tinnitus [12]. In each group the likeness matches given for 500 Hz sounds were well below the tinnitus spectrum and those for 5 kHz sounds well within it (effect of sound frequency *F*(1,26) = 58.74, *P* < 0.001) with no difference observed between the likeness matches of the groups at either frequency. Tinnitus loudness was assessed by a Borg CR100 scale (range zero to 100) and by loudness matches obtained using a 1 kHz tone (after Roberts et al. 2008) and tinnitus handicap by the THQ (total score range zero to 100). Loudness matches given by Tinn5 kHz group were higher at 1 kHz (mean = 53.9 dB SPL, see Table 1) than those of Tinn500 Hz group (*M* = 36.7 dB  SPL, *t*(26) = 2.61, *P* = 0.014), although when all matching frequencies were considered the groups did not differ from one another (*F*(1,26) = 1.13, *P* > 0.71, Figure 1(c)). Loudness ratings on the BorgCR100 scale were nonsignificantly higher in the Tinn5 kHz group (*P* = 0.16) while THQ scores were significantly worse in this group compared to the Tinn500 Hz group (*t*(26) = 2.14, *P* = 0.042). To assess whether these results suggesting a stronger tinnitus in the Tinn5 kHz group may have influenced the attention effects, pairwise correlations were calculated between tinnitus loudness matches at 1 kHz, BorgCR100 ratings, and the THQ, on one hand, and ASSR and N1 attention effects, on the other hand. The resulting correlations were directionally inconsistent and did not reach significance either in the Tinn500HZ and Tinn5 kHz groups considered separately (see Table 2) or when the two groups were combined into one sample. When passive trials only were considered, N1 TFP correlated negatively with the BorgCR100 loudness in the Tinn500 Hz group and with the THQ score in the Tinn5 kHz group reflecting lower TFP for a more disturbing tinnitus (Table 2). When the tinnitus groups were collapsed together, correlations involving tinnitus loudness measures and brain responses on passive trials were near zero and not significant. The duration of tinnitus was similar in the Tinn500 Hz and Tinn5 kHz groups (*M* = 12.5 and 11.7 years, resp., Table 1) and did not correlate significantly with the two brain responses in either group (Table 2) or when the two groups were combined.

## 4. Discussion

We previously reported that the amplitude of the ASSR (localizing to cortical sources in A1) and the N1 transient response (localizing to cortical sources in A2) was not modulated by top-down attention in tinnitus sufferers when the probe frequency was 5 kHz, a frequency known to be in the region in which tinnitus sufferers experience their tinnitus [6]. Conversely, age and hearing-threshold matched controls successfully modulated the amplitude of both responses [6] in accordance with prior evidence showing the responses to be sensitive to attention in normal hearing subjects [7, 8, 20]. It was suggested that tinnitus-related neural activity in central auditory pathways may have prevented modulation of the two responses by attention in the tinnitus sufferers. In the current experiment we tested this possibility by determining whether attention modulates these brain responses normally when evoked by a 500 Hz sound in tinnitus sufferers, which is a sound well below the TFR where tinnitus-related neural activity is believed to occur. The procedure used to assess modulation by attention was the same for the two groups, and the 500 Hz and 5 kHz datasets were combined into a single analysis which also included the long-latency auditory evoked potentials N2 and SR. We found that top-down attention modulated ASSR amplitude normally in tinnitus and control subjects probed with 500 Hz sounds and for control subjects probed with a 5 kHz sound, but not for tinnitus subjects probed with a 5 kHz sound. N1 amplitude was modulated by attention for control groups tested at each probe frequency, but modulation of N1 amplitude by attention failed for tinnitus groups tested at both frequencies. The amplitude of N2 and SR responses was modulated by attention in all groups. We discuss how attention may work in tinnitus sufferers compared to normal hearing individuals and consider how differences between these groups may be expressed in ASSR and N1 amplitude in the absence of attended performance.

### 4.1. Auditory Attention in Normal Hearing and in Tinnitus

Several lines of evidence have suggested that mechanisms that support auditory attention are persistently aroused in tinnitus [2]. One approach has been to compare the performance of subjects with chronic tinnitus with that of control subjects matched for age and verbal intelligence on cognitive tasks that require divided attention and access to memory. The rationale has been that obligatory attention to the tinnitus percept may deplete the cognitive resources needed to perform such tasks. Following this approach it has been shown that, while subjects with tinnitus perform as well as controls on tasks such as simple word naming, they do not perform as well on more complex tasks requiring retention of words in working memory over a series of sentences [22] or on Stroop tasks that divide attention between word naming and color naming [23]. The performance deficits observed in the tinnitus groups in these studies remained intact when measures of anxiety, depression, and hearing level were regressed out by covariate analyses. A more direct approach was followed by Cuny et al. [24]. In an initial demonstration based on research by Schröger [25], Cuny et al. presented normal hearing subjects with S1 stimuli in one ear that were to be ignored while they categorized S2 stimuli presented to the other (attended) ear. Performance on the S2 task was disrupted by infrequent deviant S1 stimuli, which appeared to draw attention away from the S2 task presented to the other ear. Cuny et al. subsequently found that when this task was presented to subjects with unilateral tinnitus, the interfering effect of deviant S1 stimuli was diminished when the S2 task was presented to the tinnitus ear compared to the reverse arrangement. It was suggested that persistent top-down auditory attention was directed to the tinnitus ear, such that deviant S1 stimuli presented to the nontinnitus ear could not draw attention away from it [24]. These results are in agreement with functional imaging studies of tinnitus [26, 27] which have reported increased activity in A1 and in auditory association areas that are modulated by attention when normal hearing subjects perform auditory detection tasks [2].

The presence of tinnitus did not impair behavioral performance during auditory discrimination under the conditions of our test, likely because there was no competing task requirement and most of the targets presented on the discrimination task were easy to detect. However, while ASSR and N1 responses known to be attention sensitive were modulated normally by attention in our control groups, modulation of these responses by attention was modified in tinnitus subjects. The pattern of impairment we observed could reflect differences in the functional organization of A1 and A2 and aberrant neural activity occurring in these regions in tinnitus sufferers. Unlike ASSR sources in A1 that show a frequency (tonotopic) organization in the region of Heschl's gyrus, N1 sources localize to lateral aspects of the superior temporal gyrus [18], are weakly or not tonotopic [28], and appear to reflect contributions arising from several cortical areas that comprise A2. A2 regions exhibit a heterogeneous cytoarchitectonic structure [29, 30] in which layer II/III pyramidal neurons receive inputs from diverse regions of the brain and in turn form intrinsic contacts that are more distal than in A1 where links are made in more localized modules [31]. Frequency representations which are prominent in A1 are virtually absent in A2, which appears to be specialized for processing of multidimensional auditory objects and for conveying perceptual information to higher cortical structures [30, 32, 33]. Hence it is possible that neural changes related to tinnitus (such as reduced intracortical inhibition [34], increased spontaneous activity [34, 35], and increased synchronous firing [34]) occurring in tonotopic regions of A1 may have diffusely activated A2, impairing modulation of N1 responses at both probe frequencies in tinnitus subjects. However, because A1 regions coding 500 Hz sounds are below the frequency region of A1 where tinnitus-related activity is presumed to occur, attentional modulation of the ASSR was expressed normally when tinnitus subjects were probed with this sound frequency. This interpretation is consistent with evidence from animal [1, 36] and human [37] studies which suggests that aberrant neural activity occurring in frequency regions of A1 affected by hearing impairment contributes to tinnitus percepts. It can also be aligned with previous results [38] showing that the mismatch negativity (a brain response initiated in A1 by bottom-up auditory attention, [39]) was increased in individuals with tinnitus when evoked by unexpected frequency deviants adjacent to the audiometric edge but not one octave below it. Overall it appears that persistent tinnitus-related activity occurring in the frequency region of A1 affected by hearing loss may impair modulation of the ASSR by top-down attention in this frequency region in tinnitus, but bottom-up disparities may still evoke larger responses near the lesion edge where cortical reorganization may be present [36].

Notwithstanding prior evidence for persistent auditory attention in tinnitus [24], this interpretation suggests that mechanisms of top-down auditory attention functioned normally in tinnitus sufferers under the conditions of our test, but their expression was modified by the presence of tinnitus-related neural activity occurring in central auditory pathways. Other findings of the study can be aligned with this interpretation. Subjects in the Tinn5 kHz and Cont5 kHz groups received an additional six sessions of training on the auditory detection task in the earlier study of Roberts et al. [6]. ASSR amplitude increased over training sessions in the tinnitus subjects but not in their matched controls [6] nor in previous studies using subjects with normal hearing [11, 20], possibly reflecting reduced lateral inhibition in the tinnitus subjects [1]. As training progressed, ASSR amplitude began to modulate on active blocks compared to the passive baseline in tinnitus subjects revealing an effect of attention on this response, although this modulation subsequently declined and was weak compared to that seen in controls (N1 did not modulate with attention during any session of training in the tinnitus subjects). New analyses reported in the present paper have gone further to show that the long-latency responses N2 and SR (which reach their negative maxima at central electrodes) were modulated between active and passive trials in our tinnitus groups as well as by control subjects. It is possible that these responses reflect communication between auditory regions and global networks in frontoparietal cortex that are involved in memory processing and response preparation [40]; moreover, the performance deficits cited above in tinnitus [22, 23] may derive in part from competition for resources in these pathways. In this respect we note that, while N2 and SR responses were modulated by attention in our tinnitus subjects, there was a tendency toward stronger effects in the control groups at both probe frequencies.

### 4.2. Group Differences in the Absence of Attention

Neuromodulatory systems in the basal forebrain and midbrain tegmentum are widely believed to be activated by tasks requiring attention and serve to make neurons more sensitive to their afferent input [2]. On this basis, evidence for persistent auditory attention in tinnitus could be expected to modulate the amplitude of brain responses evoked by auditory stimuli under passive conditions where tinnitus sufferers would experience tinnitus but control subjects would not. In a previous study using 40 Hz AM stimuli similar to those used here but different groups of subjects [2], we found that ASSR amplitude was larger in a tinnitus group than in controls when the carrier frequency of the probe was 500 Hz (*P* = 0.004), but this difference was reversed in groups for whom the carrier frequency was 5 kHz (*P* = 0.045). Reduced ASSR amplitude at 5 kHz was attributed to tinnitus-related synchronous activity occurring in the TFR of the tinnitus subjects (a busy line effect). Additionally, N1 amplitude was larger in the tinnitus groups compared to controls at both probe frequencies (*P* = 0.023). These results were obtained during a continuous 20-minute baseline condition in which individuals in the tinnitus groups would have heard their tinnitus. To compare these findings with the current dataset, we performed paired *t*-tests contrasting the tinnitus and control groups on passive blocks for the ASSR measured as TFP and N1 amplitude measured at electrode Fz (as in the previous work). ASSR TFP tended to be smaller in tinnitus than control subjects at 5 kHz (*P* = 0.26) and N1 larger (*P* = 0.18) at this frequency in qualitative agreement with previous results, but no group differences in ASSR or N1 amplitude reached significance in the present dataset. Overall, current evidence suggests that ASSR amplitude is larger in tinnitus subjects than in controls, at least for sounds below the TFR [2, 37]. Results regarding N1 are less consistent [2] and may reflect differences among studies with regard to the conditions of testing, stimulus procedure, and other variables that have yet to be identified.

### 4.3. Limitations and Future Directions

Within each probe frequency, our tinnitus and control groups were well matched for tinnitus characteristics, age, hearing status, and stimulus levels. Group differences in the effects of attention on brain responses at each probe frequency could not be attributed to these variables which did not differ between tinnitus and control subjects. However, while our 5 kHz and 500 Hz groups were well matched for hearing function, age range, and years of tinnitus, subjects in the 500 Hz groups tended on average to be 10 years older and their THQ scores lower than subjects in the 5 kHz groups. The intensity of the probe stimuli also differed between the 500 Hz and 5 kz conditions, in part because of the presence of threshold shifts at 5 kHz in the Tinn5 kHz and Cont5 kHz groups. To assess whether differences in these variables may have influenced our results, we correlated each variable with the effects of attention on ASSR and N1 responses at each probe frequency, collapsing tinnitus and control subjects within each frequency to increase the likelihood of uncovering alternative explanations for the findings. None of the variables correlated significantly with the effects of attention on ASSR and N1 responses, at either probe frequency. Within the limits of this analysis we conclude that differences between tinnitus and control groups in the effect of attention on ASSR and N1 amplitude reflected the presence of tinnitus in the tinnitus subjects and not the other attributes or the conditions of testing. Although interactions among different stimuli could be a limiting factor, looking forward it could be informative to modify our stimulus procedure to allow examining effects of tinnitus on attention-sensitive responses when both probe frequencies are tested within the same subjects.

A further possible limitation to consider is the extent to which a given brain response reflects the operation of an attention mechanism rather than brain processes concerned with other cognitive or behavioral functions. Active trials in our procedure required not only the deployment of attention but also the processing of target events using memory, the preparation of behavioral responses depending on target occurrence or nonoccurrence, and likely the anticipation of correctness feedback depending on outcome. As we have noted, auditory attention is known to increase ASSR amplitude when these additional factors are held constant [7], confirming the sensitivity of this response specifically to attention. Although the transient N1 response is widely believed to be sensitive to attention, as far as we are aware similar detailed analyses precluding contributions from other task features are surprisingly lacking this response. In the absence of such studies it is reasonable to assume that brain responses with short latencies are likely to reflect attention, assuming that on any attention task this process is deployed at trial onset.

Many individuals with tinnitus also experience some degree of hyperacusis expressed either by verbal reports of sensitivity to environmental sounds [41] or by loudness growth functions that are steeper than those observed in individuals with similar audiometric profiles [42]. Because we did not have a basis in the present study to distinguish between these two conditions, failure of attentional modulation could relate in principle either to the presence of tinnitus or hyperacusis or to both. It is not easy to disentangle these correlated factors in tinnitus research. However, the current findings are not easily explained in terms of altered perceptual responses to the probe stimuli in the tinnitus groups. ASSR and N1 responses might have been expected to reflect such differences under passive conditions, but the differences we observed between tinnitus and control groups were small and did not reach significance. Our practice of requiring subjects to adjust probe sound intensity to comfortable-level standard sounds presented in the frequency range of normal hearing may have attenuated effects attributable to hyperacusis in our tinnitus samples. It is also relevant that effects of attention on ASSR and N1 responses did not correlate with physical sound intensity within tinnitus and control subjects tested at 500 Hz or 5 kHz. Had perceptual responses to the probe stimuli affected attentional modulations, such correlations might have been expected but did not occur.

## 5. Conclusion

Previous studies have provided behavioral evidence of impaired performance on tasks involving control of attention in individuals with tinnitus compared to individuals without tinnitus. Our study extended the analysis to compare, between age and hearing-level matched tinnitus and control groups, the effect of attention on brain responses known to be sensitive to attention in normal hearing subjects. We focused in particular on the 40 Hz ASSR which localizes to sources in tonotopically organized primary auditory cortex (A1) and the N1 transient response which localizes to sources in nontonotopic secondary auditory cortex (A2). We found that, unlike in controls where all responses were modulated by attention, the presence of tinnitus impaired attentional modulation of the ASSR evoked by a 5 kHz but not a 500 Hz sound and the N1 evoked at both sound frequencies. We suggest that impairments of auditory attention are expressed preferentially in the 5 kHz region of tonotopically organized A1 where tinnitus-related neural activity is typically expected to occur and more diffusely in nontonotopic A2 where neuron response properties are more broadly tuned for spectrotemporal and multisensory integration.

## Figures and Tables

**Table 1:** Participant demographics.

	Tin500 Hz group	Cont500 Hz group	Tinn5 kHz group	Cont5 kHz group
*Characteristics of participants *				
Number (male)	17 (10)	16 (5)	11 (7)	11 (8)
Age in years, mean (SE)	62.0 (3.31)	62.0 (2.29)	48.6 (4.75)	53.9 (5.86)
Age range in years	22–77	42–74	22–68	22–76

*Audiometric Data *				
Mean (SE) threshold @ 500 Hz (dB HL)	10.6 (3.49)	11.9 (2.90)	8.0 (2.56)	10.5 (2.88)
Mean (SE) threshold @ 1 kHz (dB HL)	18.4 (3.76)	13.1 (3.25)	7.9 (2.70)	10.7 (3.67)
Mean (SE) threshold @ 2 kHz (dB HL)	24.5 (4.24)	12.5 (3.97)	9.8 (3.65)	13.6 (3.61)
Mean (SE) threshold @ 5 kHz (dB HL)	43.8 (5.04)	33.3 (5.19)	28.5 (6.24)	25.7 (7.01)

*Sound levels *				
Standard for matching	1 kHz pure tone 65 dB SL	1 kHz pure tone 65 dB SL	2 kHz 40 Hz AM tone at 65 dB SPL	2 kHz 40 Hz AM tone at 65 dB SPL
Mean (SE) stimulus intensity (dB SPL)	81.2 (1.68)	78.7 (2.90)	60.0 (2.05)	58.5 (2.01)
Stimulus intensity range (dB SPL)	69–93	47–93	50–74	40–66

*Tinnitus characteristics *				
Mean (SE) duration in years	12.5 (2.68)		11.7 (3.03)	
Mean (SE) loudness rating Borg CR100 scale	44.8 (5.62)		57.1 (6.21)	
Mean (SE) loudness match (1 kHz tone, dB SPL)	36.7 (9.05)		53.9 (6.32)	
THQ Mean Total Score (SE)	32.5 (5.64)		48.9 (6.66)	
Tinnitus bandwidth (number of participants)				
Tonal	12		6	
Ringing	2		2	
Hissing	3		3	
Tinnitus ear				
Bilateral	15		11	
Left	1		0	
Right	1		0	

**Table 2:** Relationship of ASSR and N1 responses on passive blocks and ASSR and N1 attention effects to subject and tinnitus variables. The table entries are product-moment correlations reported for the 500 Hz and 5 kHz conditions separately.

	Subject variables∗				Tinnitus variables			
	Age	500 Hz threshold^†^	5 kHz threshold^†^	Probe SPL	Loudness match (1 kHz)	Borg CR100	THQ	Years with tinnitus
500 Hz condition								
ASSR TFP passive	0.16	0.30	0.06	0.55^‡^	0.44	−0.03	0.44	−0.24
N1 TFP passive	0.10	0.24	−0.14	0.07	−0.25	−0.52^‡^	0.04	0.14
ASSR TFP ratio	−0.30	0.27	0.12	0.32	0.14	0.14	0.14	0.13
N1 TFP diff.	−0.28	0.06	0.05	0.02	−0.08	−0.26	0.10	0.13

5 kHz condition								
ASSR TFP passive	0.25	0.11	−0.19	−0.01	0.24	0.19	−0.40	0.05
N1 TFP passive	0.18	0.32	0.14	−0.04	0.52	0.19	−0.62^‡^	0.57
ASSR TFP ratio	−0.13	0.02	−0.20	−0.04	0.43	0.23	−0.41	0.27
N1 TFP diff.	−0.07	−0.10	−0.18	−0.06	−0.46	−0.11	0.46	−0.43

*Tinnitus and control subjects combined.

^†^Left and right ears combined.

^‡^ *P* < 0.05.

## Abbreviations

A1: Primary auditory cortex
A2: Secondary (nonprimary) auditory cortex
AM: Amplitude modulated
ANOVA: Analysis of variance
ASSR: Auditory steady-state response
DCN: Dorsal cochlear nucleus
EEG: Electroencephalogram
ITI: Intertrial interval
SPL: Sound pressure level
SL: Sensation level
SR: Auditory sustained response
TH: Threshold
THQ: Tinnitus Handicap Questionnaire
TFP: Total field power
TFR: Tinnitus frequency region.
